# EUPopLink Country report – Norway

**DOI:** 10.12688/openreseurope.23512.1

**Published:** 2026-04-24

**Authors:** Elena Baro

**Affiliations:** 1 Norwegian University of Science and Technology, Trondheim, 7491, Norway

**Keywords:** Populism, Euroscepticism, Norway

## Abstract

This country report examines the relationship between populism and Euroscepticism in Norway, a non-EU state where citizens have twice rejected EU membership yet where political and legal integration with the European Union remains extensive. Euroscepticism cuts across the political spectrum and remains tied to longstanding cleavages, especially centre–periphery divides and rural resistance to EU membership. Two populist parties—the right-wing Progress Party and the far-left Red Party—hold Eurosceptic positions, though for different reasons: the Progress Party adopts a strategic, issue-driven critique linked to immigration and sovereignty, while the Red Party advances a consistent ideological opposition to the EU as a neoliberal project undermining democratic control. The strongest Eurosceptic actor, however, is the non-populist Centre Party, which mobilizes territorial grievances more effectively than populist competitors. Norway’s consensus-oriented political culture and deliberate depoliticization of EU issues limit the political salience of European integration, making Euroscepticism fragmented and less central to populist mobilization than in many European states.

## 1. Introduction

The present country report was prepared as part of the EUPopLink COST Action ( Andreadis & Vasilopoulou, 2026) following the EUPopLink Theoretical Framework and guidelines ( Lorimer et al., 2026).

As a non-EU country whose citizens narrowly rejected EU membership twice—in the 1972 and 1994 referenda—Norway has developed a reputation for Euroscepticism. Despite these rejections, the country has established extensive legal and political cooperation with the EU. This relationship includes signing the European Economic Area (EEA) Agreement and active participation in justice and home affairs, foreign, security, defence policy ( Græger and Haugevik, 2022), as well as energy and climate.

Through the EEA, Norway enjoys access to the EU’s internal market yet remains outside the EU’s institutional decision-making processes. Consequently, the EU’s presence in Norway manifests primarily through adopted regulations and legal alignment, which are less apparent to most citizens compared to EU member states, where integration may be perceived more concretely by citizens and have more visibility in public discourses ( Hansen et al., 2025).

While Norway has low polarization levels (see Bein, 2025), the EU membership question remains notably divisive, and has been one of the most politically divisive issues in the country ( Fossum, 2020). On the basis of such divisiveness is the fact that the EU question brings (back) to the table a multitude of issues ranging from longstanding yet still active political cleavages—especially the centre–periphery and urban–rural divides—to economic concerns, party dynamics, and cultural values (e.g., Solli, 2020). The centre–periphery cleavage is particularly relevant in this context and sees a pro-EU political centre in Oslo juxtaposed against more Eurosceptic peripheral regions in the West and North, particularly rural communities reliant on farming and fishing, which remain consistently against EU membership ( Fossum, 2020). In terms of party dynamics, conflicts related to the EU question are not only between ‘yes’ and ‘no’ parties but have also involved significant internal divisions within major parties, such as the Labour Party. Regarding economic and cultural arguments, the former highlight concerns that EU membership could threaten Norway’s welfare state, disrupt the domestic labour market, weaken environmental protections, and offer limited economic benefits—especially given Norway’s substantial oil revenues—while the latter include concerns about preserving Norwegian cultural identity and national sovereignty.

These concerns cut across the political spectrum and remain relevant in both party politics and public opinion. Nevertheless, since the referenda, Norwegian political parties have largely kept the issue of European integration off the national political agenda. This silence stems from an implicit agreement across parties not to reignite debates on EU membership or the lack of democratic accountability associated with the EEA Agreement ( Gänzle and Henökl, 2017). The EU question has been described as a political “suicide clause” ( Fossum, 2008), having historically triggered the fall of several coalition governments due to internal divisions ( Sitter, 2001). The proportional electoral system, which typically involves the formation of coalition governments, complicates things even further, as coalitions frequently include both pro- and anti-EU parties, making agreement on European integration especially challenging ( Fossum, 2020).

Overall, this “silent agreement” not to bring back the EU membership issue is also in line with relatively stable trends in public opinion, which see a slight but consistent majority of Norwegians opposing EU membership. According to a recent NUPI survey ( Svendsen 2024), support for the EEA Agreement itself remains relatively moderate, with 43% in favour, 33% who oppose it, and a 24% who remain undecided. When it comes to full EU membership, however, although support has slightly increased—from 25% in 2021 to 31% today—the majority (38%) still prefers a less comprehensive arrangement with the EU. Nevertheless, recent events, particularly Russia’s invasion of Ukraine, have partly renewed debates on EU integration, leading some to advocate closer ties with the EU, as well as impacting public opinion on the topic. The war in Ukraine has somewhat influenced perceptions of EU membership’s relevance: 45% of Norwegians feel the war has increased the relevance of joining the EU, while 37% disagree, with differences being often explained by education level, income, and political preferences, with voters of the right-wing populist Progress Party being more likely to express opposition ( Svendsen 2024).

Additionally, as considerable time has passed since the last referendum in 1994, there are growing calls for the need of a new referendum, emphasizing that many Norwegians today have never had a direct say on EU membership. Despite these calls, however, continued public division and uncertainty deter political parties from reintroducing the topic onto the political agenda.

## 2. Populist political actors

Norway has a modest presence of populist parties, with the most electorally successful one being the right-wing populist Progress Party ( *Fremskrittspartiet*), and a case classified as borderline populist party (see Rooduijn et al., 2023), positioned on the far-left-side of the ideological spectrum, the Red Party ( *Rødt*). Both parties are currently Eurosceptic.

• Progress Party: the party was founded in 1973 as an anti-tax party and has successively gained increasing electoral support from the 1980s, when it took ownership of the immigration issue, politicizing it. The party has been part of a governing coalition between 2013 and 2020. The main policy and issues the Progress Party advocates are strict regulations for immigration, tax reduction, increased use of the revenues of the Norwegian State Oil Fund and overall neoliberalist policies ( Bjånesøy, 2023; Jupskås, 2016). Even though immigration remains the most prominent and important issue in the Progress Party’s agenda, the party remains rather moderate compared to other PRR parties, particularly due to the lack of prominent nativist positions. Perhaps also considering the status of Norway as a non-EU member state, the Progress Party has generally avoided formal cooperation with other populist parties in Europe, preferring a more independent approach. Despite ideological similarities, the party has resisted joining formal populist groups like the Nordic Freedom alliance in the Nordic Council due to policy differences and strategic considerations ( Jupskås, 2016).
• The Red Party is a far-left borderline populist party which emerged in 2007 from the merger of several far-left groups, including the Red Electoral Alliance (RV) and the Marxist-Leninist AKP, bringing forward a legacy of anti-capitalist activism and strong EU scepticism ( Bjørklund & Fjeldavli, 2023). The party has its roots in class politics and a consistent focus on economic redistribution, welfare protection, and opposition to neoliberalism. It endorses a revolutionary ideology and supports the idea of a classless society, issues which are often promoted using a populist rhetoric to appeal to its electoral base. In recent years, the Red Party has gained momentum, particularly among working-class voters, by prioritizing issues such as social inequality, labour rights, and public sector investment. The party’s 2021 electoral results represented its best results ever (4.7%) and were largely driven by its ability to mobilize support around material issues like redistribution and welfare, combined with a critique of the political establishment ( Bergh & Haugsgjerd, 2023). While the Red Party is not part of any European parliamentary group, it cooperates informally with other Nordic leftist parties through the Nordic Green Left Alliance.

## 3. Positions on ‘Europe’ and the populism-euroscepticism nexus

Euroscepticism in Norway is quite diffuse across the political spectrum and not limited to populist parties. The two main parties classified as populists which also currently hold Eurosceptic positions are the Progress Party and the Red Party. Remarkably, the Centre Party ( *Senterpartiet*) is probably the Norwegian political party advocating the strongest Eurosceptic sentiments and having the closest connection to the topic. Consequently, it has been included in the current section of the analysis, even though – while it often frames its position on Europe in a people versus elite fashion – it has not been officially classified as populist.

The Progress Party distinguishes itself from many of its European right-wing populist counterparts through its comparatively ambiguous stance on European integration ( Taggart and Pirro 2021). Historically, the Progress Party adopted varied and at times conflicting positions on the EU. In the 1980s and early 1990s, the party expressed support for Norwegian EU membership, as it aligned with the party’s economically liberal agenda and emphasis on market deregulation ( Sitter, 2001). This initial, more positive phase has been followed by a period marked by internal disagreements, and the issue’s divisiveness led the party to maintain a low profile on the matter for years. This changed in 2016, when the party officially declared its opposition to EU membership, becoming the first party on the political right in Norway to do so explicitly ( Solli 2020). The party justified its hard Eurosceptic stance by arguing that the EU had shifted from a peace-oriented project to a source of internal conflict among member states. In the years that followed, the Progress Party’s Euroscepticism became more pronounced and articulated, and it began to align more clearly with its nationalist, anti-immigration platform. The party has voiced opposition to Schengen and supported limiting welfare provisions to Norwegian citizens. However, despite the critiques it raises, the party remains unlikely to initiate significant institutional change ( Fossum, 2020) and continues to avoid using the issue prominently in electoral campaigns ( Solli, 2020).

While the Progress Party’s voters display relatively low levels of trust in the EU—second only to those of the Centre Party— the partisan divide on EU attitudes in Norway is less pronounced than in other Scandinavian countries and is probably explained by the lower salience that the EU issue has in non-member states like Norway ( Hansen et al. 2025).

An additional dynamic that might help explaining the less prominent role of Euroscepticism for the populist Progress Party is that the territorial framing of Euroscepticism—a particularly relevant line of conflict involving centre–periphery divides and rural resistance to EU membership and integration—has been more consistently and effectively politicized by the Centre Party. The Centre Party has been steadily and vocally critical not only of EU membership but also of the EEA Agreement itself. Its position reflects a form of *hard Euroscepticism*, grounded in concerns about sovereignty, democratic accountability, and rural autonomy ( Skinner 2010). Although it does not systematically frame its opposition to European integration in classic populist terms, the Centre party’s discourse frequently features antagonistic narratives that position “ordinary people,” especially rural and agricultural communities, against out-of-touch elites in both Brussels and Oslo ( Skinner 2011). This may explain why the Progress Party’s Euroscepticism, while present, remains less ideologically central to its platform compared to similar parties elsewhere in Europe ( Sitter, 2001; Solli, 2020).

Interestingly, the far-left-wing (borderline) populist Red Party has—unlike its right-wing counterpart—maintained a consistent and hard Eurosceptic stance over the years. The predominance of the Red Party’s position became particularly visible in response to Brexit. Given Norway’s status as a non-member, some expected Brexit to trigger similar anti-EU sentiments. Although some political actors called for changes—ranging from renegotiating the EEA Agreement to Schengen and trade agreements—there was little coordination in these demands. Moreover, the parties most vocal about changing Norway’s European affiliation were not on the populist right, but on the far left. The Red Party expressed criticism of social dumping and labour mobility but did so from a redistributive and egalitarian perspective rather than a nationalist or anti-immigration one, as in the case of Brexit. Their vision of sovereignty and economic justice was ideologically far removed from the UK Conservative Party’s model and instead reflected a clear commitment to a more leftist socioeconomic alternative ( Fossum & Vigrestad, 2021). As per party manifesto, the Red Party opposes both EU membership and the EEA Agreement, arguing that European integration erodes democratic self-rule and prioritizes market liberalism over social justice. They argue that the EEA Agreement has led to Norway being subject to EU legislation without having a say in its formation, which undermines Norwegian sovereignty — a line of reasoning also echoed by the Progress Party and the Centre Party in debates on Norwegian energy prices. In response, the Red Party advocates for replacing the EEA Agreement with bilateral trade deals and stresses the importance of actively using Norway’s right of veto within the current framework until such replacements are established. Its critique targets both the institutional design of the EU and the neoliberal policy agenda it promotes, which they claim limits Norway’s ability to uphold redistributive policies, labour rights, and public services. The party rhetoric often frames the EU as a project imposed by economic and political elites, which is in line with a left-wing populist narrative that positions the Norwegian people against supranational economic elites.

In addition to these parties, other non-populist political actors express forms of hard Euroscepticism. The Christian Democratic Party ( *Kristelig Folkeparti*) opposes full EU membership but endorse political and economic collaboration with the EU when it aligns with Norway’s national interests. The party support the existing framework of engagement, primarily through Norway’s participation in the European Economic Area (EEA). In contrast, the Socialist Left Party ( *Sosialistisk Venstreparti*) has, like the Centre Party, consistently rejected formal institutional ties with the EU. Instead, it advocates for cooperation based on the original Free Trade Agreement ( Leruth, 2017).

## 4. Responses to euroscepticism and consequences

The Norwegian case is rather peculiar when it comes to discussing the responses to Euroscepticism of mainstream parties. The reasons are the divisiveness of the EU topic, coupled with the consensus-oriented political system. Lastly, Euroscepticism is widely diffuse across the ideological spectrum —even though to varying degrees— and not limited to populist or challenger parties.

Currently, the pro-EU mainstream parties in Norway include the Conservative Party ( *Høyre*), the Labour Party ( *Arbeiderpartiet*), the Liberal Party ( *Venstre*), and the Green Party ( *Miljøpartiet De Grønne*). As noted, the Labour Party was previously divided on the EU issue, particularly around the referenda. The Liberal Party’s support for EU membership is also relatively recent, having reversed its long-standing opposition in 2020. Likewise, the Green Party shifted its position in 2023, moving from opposition to endorsing a new referendum and stronger EU ties—especially in light of climate policy priorities and recent geopolitical developments.

The Conservative party has long held a pro-EU membership stance ( Leruth, 2017). The party manifesto’s principles explicitly call for pursuing full EU membership, emphasizing that such integration secures jobs, welfare, and influence over EU decision-making. The party leader Erna Solberg, reaffirmed the Conservative’s EU-supportive orientation, stating explicit support for EU membership and backing immediate accession if political conditions allow. On a slightly different note, the Labour Party retains a consistently pro-European stance but holds a cautious position on full EU membership — partly due to the Eurosceptic positions of the Norwegian Confederation of Trade Unions (LO), with which it maintains historically close ties. However, recent developments—including the collapse of the Centre Party coalition over adoption of EU energy directives in January 2025—have reignited internal discussions about membership. While the party leadership maintains that “now is not the right time”, an increasing number of parliamentarians support revisiting the question.

Regarding Norwegian parties’ response to Euroscepticism, one may at first sight argue that the most common strategy has been a ‘dismissive’ one as in Meguid (2005). A closer look will however provide us with a slightly different picture. Specifically, all Norwegian political parties across the political spectrum have made more or less tacit agreements not to discuss the EU membership issue and to an overall toning down of EU discussions, including the legislative integration process (see Fossum 2008). Norwegian governing coalitions, in the light of the contentiousness of the issue and of the so called ‘suicide clauses’ —entailing that if a partner launches a campaign for EU membership, the coalition unravels ( Fossum 2008)— have kept the issue at bay and off the political agenda. The overall purpose has been to ensure agreement and consensus, and to prioritise the well-functioning of Norwegian democracy ( Fossum 2008). As anticipated, following the Russian invasion of Ukraine, and the recent 30 years anniversary of the last EU referendum, the question of Norwegian EU membership has recently re-entered public debate. Though no formal steps toward a referendum have been initiated, the convergence of uncertainty, institutional critique, and generational change has reenergized the EU debate in both media and political discourse.

Likely due to the diffuse nature of Euroscepticism across political parties and deliberate efforts to keep the issue low on the public and political agenda, the consequences of Norwegian populist parties’ Euroscepticism have remained relatively modest. On the one hand, the Progress Party adopts a more strategic, issue-driven and situational form of Euroscepticism, deploying anti-EU rhetoric primarily in relation to issues like immigration and sovereignty, but supporting —at least partially— the EEA framework and rarely centring Europe in its policy agenda ( Skinner 2010; Bergh & Haugsgjerd 2023). In contrast, the Red Party’s opposition to both EU membership and the EEA agreement is more ideologically consistent, rooted in a structural critique of the EU as a neoliberal project that undermines democratic control and public welfare. Yet due to its marginal influence in national policymaking, the Red Party’s position has had limited practical impact. Unlike the Centre Party, which exited government in January 2025 over EU energy directives, neither the Progress Party nor the Red Party have translated their Euroscepticism into concrete action, suggesting that for these actors, the EU question remains symbolically important but politically peripheral.

## 5. Short conclusion

To conclude, Euroscepticism in Norway is a diffuse phenomenon, that cuts across the political spectrum and moves beyond populist parties. Its relationship with populism is distinctive, and arguably less central than in many other European contexts. Instead of being monopolized by populist parties, Eurosceptic positions are embedded within the broader ideological frameworks of diverse political parties. Notably, the non-populist Centre Party has been particularly effective in mobilizing the territorial dimension of Euroscepticism, drawing on deeply rooted rural and centre–periphery divides. Additionally, Norway’s position as a non-EU member state reduces the political salience of EU-related debates. Considering the political culture oriented toward consensus, as well as the historically polarizing nature of the EU issue, Norwegian parties have strategically opted to keep EU-issues off the agenda. All these factors have likely contributed to limiting the political space for populist parties to claim sole ownership over the EU issue, as well as making Euroscepticism, while present and politically significant, less prominently mobilized and more fragmented than in other contexts.

## Ethics and consent

Ethical approval and consent were not required.

## Data Availability

This article does not rely on any dataset.
