# Supplementary material for: EUPopLink Country report – Norway
Source: Open Res Eur. 2026 Apr 24;6:133. [Version 1] doi: 10.12688/openreseurope.23512.1 (PMC13146460; doi:10.12688/openreseurope.23512.1)
Supplement: Peer review history

# Reviewer report: Reviewer response for version 1

**DOI:** 10.21956/openreseurope.25453.r73221

**Published:** 2026-05-22

**Authors:** Roch Dunin-Wąsowicz

**Affiliations:** 1 University College London, London, UK

This report that captures the specificity of the Norwegian case, particularly the paradox of extensive integration without EU membership and the resulting diffuse and low-salience character of Euroscepticism. The discussion of how Euroscepticism cuts across party lines is especially convincing. The report also provides a nuanced differentiation between strategic and issue-contingent Euroscepticism, as well as ideologically consistent opposition to the EU.

 The article would benefit from a more explicit and theoretically grounded definition of populism. While parties are classified as populist or borderline populist, the criteria underpinning these classifications remain somewhat implicit. Relatedly, the report convincingly shows that Euroscepticism in Norway is not monopolized by populist actors. However, without a sharper conceptualization of populism, it becomes more difficult to fully appreciate the extent to which the observed patterns reflect a linkage between populism and Euroscepticism.

 Another strong element of the report is its attention to the territorial dimension of populist Euroscepticism. This dimension could be developed further by moving beyond party strategies and considering the structural conditions that underpin the salience of territorial Euroscepticism. Recent contributions suggest that factors such as economic geography, sectoral dependence (e.g. agriculture and fisheries), and uneven exposure to globalization shape the demand-side foundations of Euroscepticism. Incorporating this perspective would allow the analysis to better explain why territorial grievances persist and why they are more effectively mobilized by certain actors, rather than treating them primarily as products of political agency.

 Overall, the report makes a valuable empirical contribution and offers a nuanced account of a relatively understudied case. Strengthening the conceptualization of populism and further developing the structural underpinnings of the territorial dimension would enhance its theoretical leverage and make its findings more directly comparable to the broader literature on populism and Euroscepticism.

Is the case presented with sufficient detail to be useful for teaching or other practitioners?

Yes

Is the work clearly and accurately presented and does it cite the current literature?

Yes

If applicable, is the statistical analysis and its interpretation appropriate?

Not applicable

Are all the source data underlying the results available to ensure full reproducibility?

No source data required

Are the conclusions drawn adequately supported by the results?

Yes

Is the background of the case’s history and progression described in sufficient detail?

Yes

Reviewer Expertise:

Dr Roch Dunin-Wąsowicz is Associate Professor of Sociology, Politics and Public Policy at UCL Social Research Institute, University College London, and Research Associate at the Conflict and Civicness Research Group at LSE IDEAS. His scholarship centres on migration, civil society, and the civic engagement of business, with a track record of peer-reviewed publications on Brexit, populism, and transnational support for Ukraine. He has led numerous high-impact, collaborative projects producing influential research and policy outputs. His strategic leadership bridges research and advocacy, shaping public policy, enhancing academic inclusivity, and mentoring future scholars across diverse disciplines and regions. His current research interests cover transnational entrepreneurs and businesses working towards civic end-goals at the frontlines of conflict.

I confirm that I have read this submission and believe that I have an appropriate level of expertise to confirm that it is of an acceptable scientific standard.

## References

[1] : Bridging the Divide Between Structural and Actor-Oriented Explanations of Populism: A Research Agenda for the Study of Populist Euroscepticism Through a Territorial Perspective. *Political Studies Review* .2025;23(2) : 10.1177/14789299241296796 622-634 10.1177/14789299241296796

[2] : Geographical Dimensions of Populist Euroscepticism. *Political Studies Review* .2025;23(2) : 10.1177/14789299231201810 678-689 10.1177/14789299231201810

# Reviewer report: Reviewer response for version 1

**DOI:** 10.21956/openreseurope.25453.r73219

**Published:** 2026-05-05

**Authors:** Anna Kyriazi

**Affiliations:** 1 University of Milan, Milan, Italy

This case study examines Euroscepticism in Norway. Norway is interesting because it is not a full member state of the EU, but nonetheless cooperates extensively through the EEA Agreement. The report shows that Euroscepticism is widespread across the political spectrum. While populist parties like the Progress Party and the Red Party hold Eurosceptic views, the strongest anti-EU stance comes from the non-populist Centre Party. EU issues remain overall low-salience in national politics despite being polarizing.

 While the report is fundamentally sound, I of course have some feedback that may be considered for further improving the text:

 Since many of the report’s readers will be unfamiliar with the Norwegian context, the report would benefit from adding a few more details and explanations on key issues, including the following: Why are rural communities the most anti-EU constituency in the country? What is the background to the government’s collapse over EU energy directives? This seems very significant but is not explained in the text.

 Finally, scattered throughout the report are clues as to when the EU issue -- and with it Euroscepticism -- flares up in a context of generally low salience. Brexit, the anniversary of the membership referendum, and Russia’s war on Ukraine are mentioned. Perhaps these events deserve a more systematic treatment, as they seem to provide key opportunities for populist mobilization and realignment.

Is the case presented with sufficient detail to be useful for teaching or other practitioners?

Yes

Is the work clearly and accurately presented and does it cite the current literature?

Yes

If applicable, is the statistical analysis and its interpretation appropriate?

Not applicable

Are all the source data underlying the results available to ensure full reproducibility?

No source data required

Are the conclusions drawn adequately supported by the results?

Yes

Is the background of the case’s history and progression described in sufficient detail?

Yes

Reviewer Expertise:

comparative European politics

I confirm that I have read this submission and believe that I have an appropriate level of expertise to confirm that it is of an acceptable scientific standard.

# Reviewer report: Reviewer response for version 1

**DOI:** 10.21956/openreseurope.25453.r73218

**Published:** 2026-05-04

**Authors:** Simon Usherwood

**Affiliations:** 1 The Open University, Milton Keynes, UK

The piece provides a succinct overview of Norwegian euroscepticism, focusing on populist parties, supported by wider context. While it is clear that populism is not the biggest driver of euroscepticism in the country, it would be interesting to read more about the interplay between the two, since this would highlight further the need for a differentiated analytical and practice response to the two phenomena. However, this is perhaps more a point for subsequent development: as it stands, the report is clearly and logically presented, making good use of source materials and with clear conclusions.

Is the case presented with sufficient detail to be useful for teaching or other practitioners?

Yes

Is the work clearly and accurately presented and does it cite the current literature?

Yes

If applicable, is the statistical analysis and its interpretation appropriate?

Not applicable

Are all the source data underlying the results available to ensure full reproducibility?

No source data required

Are the conclusions drawn adequately supported by the results?

Yes

Is the background of the case’s history and progression described in sufficient detail?

Yes

Reviewer Expertise:

Euroscepticism, UK-EU relations

I confirm that I have read this submission and believe that I have an appropriate level of expertise to confirm that it is of an acceptable scientific standard.
